# The Force Cone Method Applied to Explain Hidden Whirls in Tribology

**DOI:** 10.3390/ma14143894

**Published:** 2021-07-13

**Authors:** Claus Mattheck, Christian Greiner, Klaus Bethge, Iwiza Tesari, Karlheinz Weber

**Affiliations:** 1 Institute for Applied Materials (IAM), Karlsruhe Institute of Technology (KIT), Kaiserstrasse 12, 76131 Karlsruhe, Germany; mattheck@web.de (C.M.); klaus.bethge@kit.edu (K.B.); iwiza.tesari@kit.edu (I.T.); karlheinz.weber@kit.edu (K.W.); 2 KIT IAM-CMS MicroTribology Center (µTC), Strasse am Forum 5, 76131 Karlsruhe, Germany

**Keywords:** tribology, vortices, force cone method, whirls, FEM

## Abstract

In tribologically loaded materials, folding instabilities and vortices lead to the formation of complex internal structures. This is true for geological as well as nanoscopic contacts. Classically, these structures have been described by Kelvin–Helmholtz instabilities or shear localization. We here introduce an alternative explanation based on an intuitive approach referred to as the force cone method. It is considered how whirls are situated near forces acting on a free surface of an elastic or elastoplastic solid. The force cone results are supplemented by finite element simulations. Depending on the direction of the acting force, one or two whirls are predicted by the simplified force cone method. In 3D, there is always a ring shaped whirl present. These modelling findings were tested in simple model experiments. The results qualitatively match the predictions and whirl formation was found. The force cone method and the experiments may seem trivial, but they are an ideal tool to intuitively understand the presence of whirls within a solid under a tribological load. The position of these whirls was found at the predicted places and the force cone method allows a direct approach to understand the complex processes in the otherwise buried interfaces of tribologically loaded materials.

## 1. Introduction

When metals are subjected to a tribological load, the same basic materials science principle applies as for other forms of mechanical loading: The microstructure of the material determines its properties. For a frictional contact however, this concept is slightly more complicated, as the microstructure directly at and under the contacting surfaces undergoes highly dynamic changes due to the shear loading [1,2,3,4,5]. The intricate interplay and feedback between surface stresses, subsurface grain size and friction coefficient was recently elucidated by Argibay and co-workers [6]. These phenomena are of immense interest, as in metals the frictional energy is mainly dissipated through plastic deformation and microstructural changes [7]. In metals under a tribological load, vortices, waviness and folding instabilities are often observed as microstructural characteristics [8,9,10]. In the literature, the formation of such microstructural features has been attributed to several mechanisms. For example, the plastic response of the material has been treated like a fluid [9,11]. Doing so leads to structures reminding of Kelvin–Helmholtz instabilities [8,9,10,11]. For very similar features that originated during severe plastic deformation experiments conducted with copper, silver and aluminum foils, vortices were explained by treating these materials as non-linear viscous fluids [12]. This again leads to instabilities due to shearing which are accommodated by the formation of vortices [12]. More recently and for metallic materials, crystal defects as well as their ability to accommodate slip on certain glide systems was identified as the main reason behind whirl formation in multilayer systems [13]. As was pointed out by Rigney et al. [11] and Pouryazdan et al. [12], vortex formation is observed over a wide range of length scales [14]: In nanoscopic molecular dynamics simulations [10], over the micrometer length scales of tribological model systems [8], the millimeter scale in high pressure torsion experiments [12], as well as in geology [14]. This demonstrates the generality of this phenomenon as well as its tremendous importance when it comes to understanding a material’s response to a shear load. The same is true when looking at recent molecular dynamics results for the subsurface microstructure evolution of CuNi alloys in a tribological contact. In addition, there signs for whirl or vortex formation can be found [15]. Here, we offer an alternative and intuitive view for explaining whirl formation on a continuum level. This approach is based on the established force cone method, which was initially inspired by tree architecture [16,17,18]. This approach was in the past successfully applied in industry to design lightweight and durable components, i.e., by casting and forging [19]. The force cone approach has the distinct advantage of being descriptive and thereby easy to apply. At the same time one needs to keep in mind that microstructural elementary processes like dislocation motion or grain boundary sliding by which a material will accommodate these forces [6] are of no concern to the force cone method. This in turn is the advantage of the approach; it can be applied to a wide range of materials, as the exact mode of shear accommodation does not matter to a mesoscopic, continuum explanation.

## 2. Materials and Methods

### 2.1. The Force Cone Method

**Figure 1:**
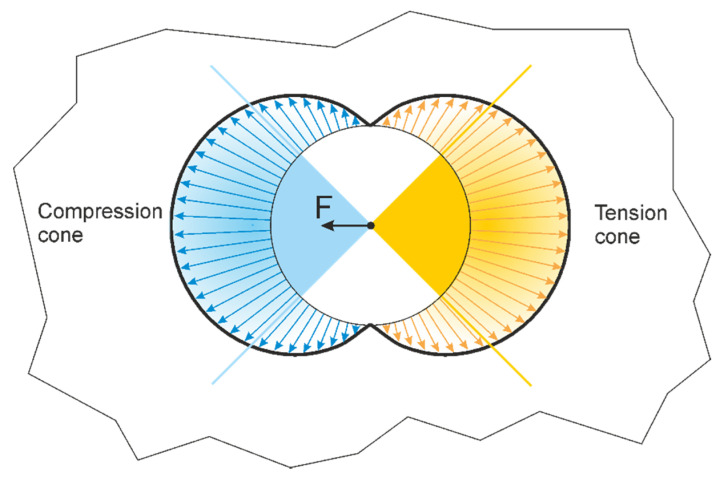
Distribution of radial stresses in an infinite plate loaded by a single force and superposition of force cones [18] and reproduced with permission.

The basic idea behind the force cone method is that a single force acting at a point in an infinite elastic plane pushes a compression cone ahead and pulls a tension cone behind. Figure 1 shows the distribution of radial stresses at a distance, r = 1, from the point of force application in an infinite plate loaded by a single force [20]. The arrows indicate the magnitudes and directions of the compressive (blue) and tensile (yellow) stresses. Superposition with the appropriate force cones indicates that most of the stresses act within the two 90° sides of the force cones. The 90° force cone assumption thus served to eliminate less loaded regions right before the final shape is determined and used for designing lightweight structures.

**Figure 2:**
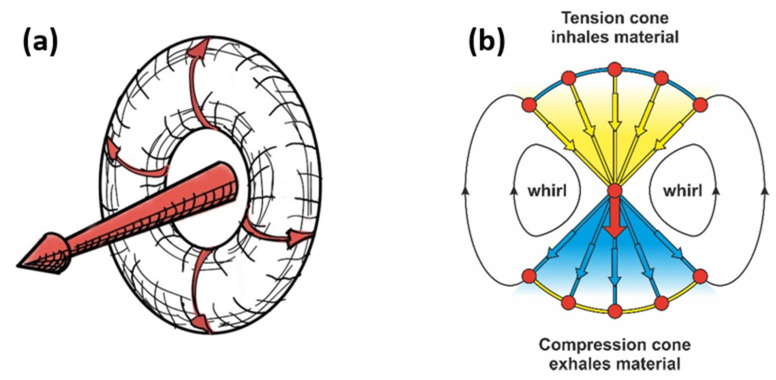
About the force cone method. (**a**) Every force in a solid creates a rotating displacement field in the form of a ring whirl. There is a field of very small elastic displacements, which altogether describe the shape of the whirl. The 2D model in (**b**) is a cross-section along the diameter of the overall whirl running through the force axis. The red arrow is the force attacking at the center (red dot). The compression cone is colored in blue; the tension cone is in yellow. The red dots show some of the intersection points of compressive and tensile force flow (principle stresses) which cross each other perpendicularly. The blue arch in the tension cone is one of the many compression bows. The yellow arch in the compression cone is one of the many tension “cords” related to the principle tensile stresses.

The principle of the force cone method and whirls in solid materials is highlighted in Figure 2.

Any force acting inside a solid pushes a compression cone in front and pulls a tension cone behind itself [16]. If one imagines that the compression cone is filled with compacted material, it “exhales” excessive material. Simultaneously, this material can be “inhaled” by a tension cone of another force nearby. These processes are comparable to the material flow from a source to a sink.

In Figure 2, the tension cone is the consequence of the same force as the compression cone, but the material can also be inhaled by the tension cone of any other force nearby. With this approach, the force cone method has established itself as an easy to grasp concept to perform topology optimization for creating light weight structures without computer effort as well as to extend the lifetime of engineering structures [16,17].

**Figure 3:**
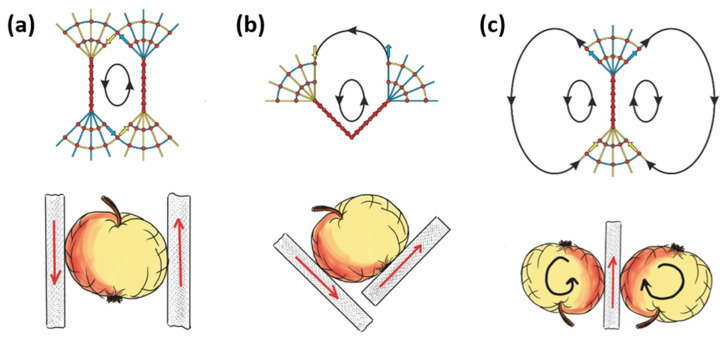
Force cone method and different generalized loading scenarios. Combinations of line loads with the related blue compression cones and yellow tension cones. Drawing a line from the blue compression cone to the nearest yellow tension cone results in the whirl shaped displacement field. The intensity of the whirl’s drive is decreasing (**a**–**c**), i.e., it decreases with the distance from the compression cone to the tension cone. In the lower images, the whirls are visualized by rotating apples. Images adapted from [16] and reproduced with permission.

In order to exemplify further how the force cone method can be an intuitive predictor for the localization of whirls in solids, and for a combination of different acting loads, three further examples are presented in Figure 3.

In the three examples of line loads highlighted in Figure 3, the magnitude of the acting forces can be increased. When allowing for plastic flow, the force cone method is able to qualitatively predict the shape of the red-colored plastic yield zones qualitatively, whilst their extent and size depends on the material properties, like yield stress, strain hardening coefficient, and most importantly the magnitude of the external load.

**Figure 4:**
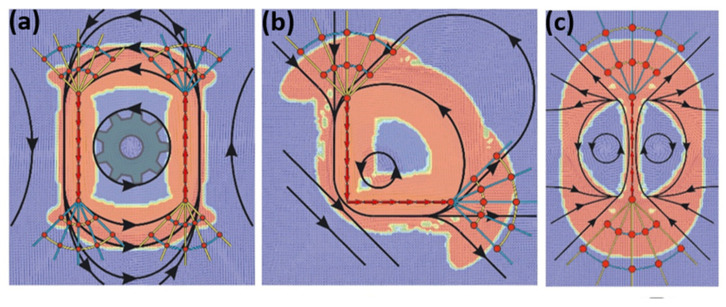
FEM results for whirls and plastic yield zones. For the line loads introduced in Figure 3**a**–**c**, finite element calculations were conducted here (**a**–**c**). As predicted by the force cone approach, the plastic yield zones extend from the compression cone to the next tension cone and along the line load. The shape of the red plastic yield zones is correlated to the whirls. Smaller loads give open plastic rings, which coalesce when the load is increased. The black solid lines interconnect the individual displacement vectors. Images taken from [16] and reproduced with permission.

For the same loading conditions considered in Figure 3, elastoplastic finite element method (FEM) calculations were conducted, with the results presented in Figure 4. For these FEM calculations, a generic elastoplastic material model was employed.

### 2.2. Application of the Force Cone Method to a Single Point Surface Load

**Figure 5:**
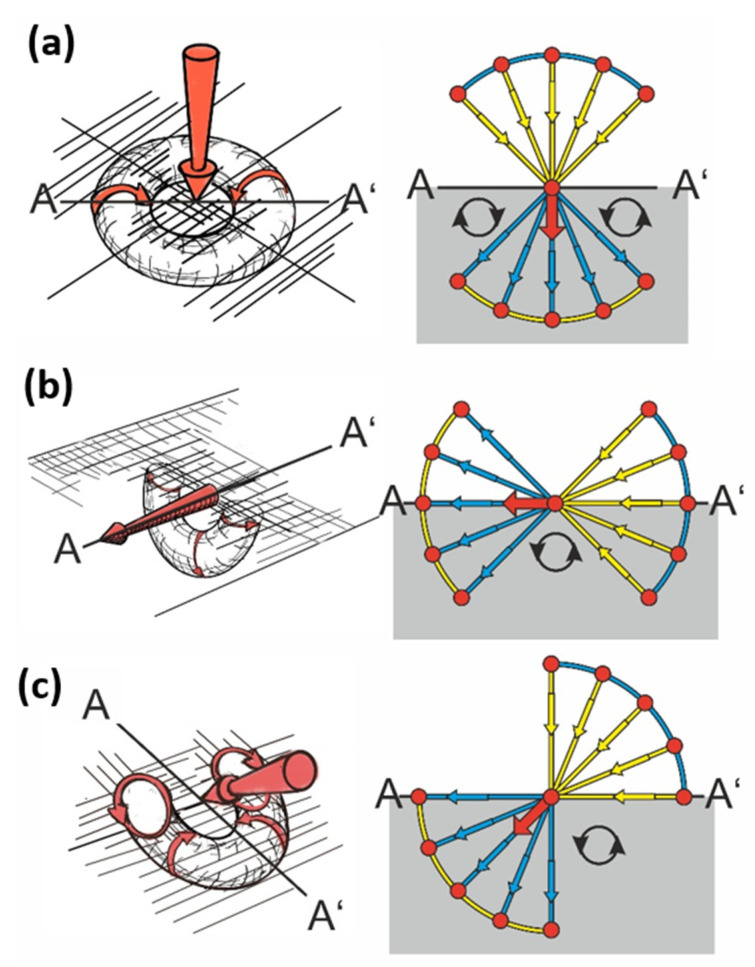
The force cone method applied to point surface forces. In order to simplify a tribological loading scenario, the surface forces acting on a single point at the surface are considered (**a**) perpendicular to the surface; (**b**) parallel to the surface and (**c**) at an angle of 45°. In all cases, the red arrow in the left sketches represents the acting force. Force cone drawings on the right represent the cross-sections when cutting the solid at the A-A’ axes. This approach is intended to allow for a qualitative assessment of the whirl situation near the forces.

As a first simplification, a tribological load is reduced to a force acting on a single point on a solid’s surface. Applying the force cone method allows for a first qualitative intuition where vortices are expected to form at or under the surface. The results are presented in Figure 5.

The force cone cross-sections in Figure 5 demonstrate that the force cone method does allow to develop a first, qualitative sense for where whirls might be expected under a tribological load. For a pure indentation (Figure 5a), where the force is acting perpendicular to the surface, a whirl ring is found. In gases similar phenomena are known, for example the rings created by cigar smokers. Interestingly, a similar whirl was predicted by Prandtl [21,22] when he was studying slip lines under a vertical localized pressure acting on an elastoplastic half space. In soil mechanics such whirls are often discussed when houses fail on wet soil [23]. Assuming that two solids are sliding against each other, one might simplify the acting forces to a single force parallel to the surface. This scenario is presented in Figure 5b. A half-ring whirl develops and the force cone drawing on the right illustrates the situation at the plane of symmetry. For a force at an angle of 45° to the surface normal (Figure 5c), only the compression cone is fully inside the solid. The tension cone is loading the surface merely tangentially. Therefore, along the plane of symmetry A-A’ only one whirl is to be expected inside the solid and the three-dimensional sketch on the left suggests two whirls at the surface.

### 2.3. Finite Element Method

As intuitive as the force cone method might be, it is based on severe simplifications. It might therefore be instructive to compare the force cone to FEM results. We chose FEM for all three loading scenarios and in some cases already established analytical solutions following the equations developed by Boussinesq [24]. Classic elastomechanics solutions [20,25] for the well-known Kelvin’s problem, a point-loaded infinite body, also contain whirls which can be found by visualizing the displacement fields. In addition to the classic solutions, FEM allows us to study an elastic-plastic contact. For doing so, the following 2D finite element method calculations were performed with the finite element program ANSYS Mechanical (Canonsburg, PA, USA), Version 17 through the Ansys Parametrical Design Language (APDL) [26]. Simulation parameters are listed in Table 1. The material parameters are somewhat arbitrary and not meant to model the behavior of a specific material in particular. Especially the low-yield stress and low plastic tangent modulus were chosen in order to allow for an early plastic response, large yield zones and pronounced plastic whirl displacements, even for small loads. It should also be pointed out that we chose point loads—distributed over three nodes—and not contacts of finite sizes in order to keep our FEM analysis simple and to allow for an easy comparison with the force cone results, where also a point load is considered.

**Figure 6:**
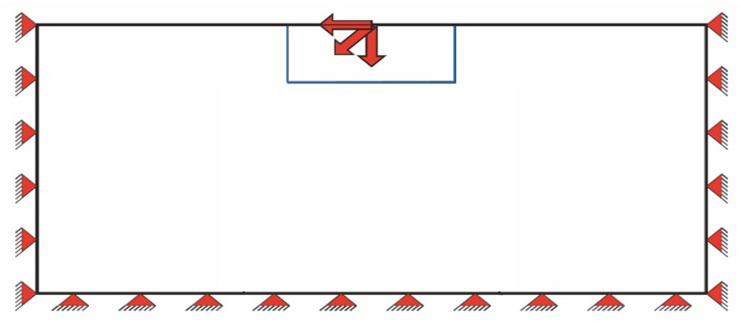
FEM set-up. An elastoplastic material is fixed at the sides and at the bottom. At the free surface a single point force is acting on the solid at 0°, or 45°, or 90° to the surface normal.

As boundary conditions, the FE model was fully clamped at three sides with only the upper side as a free surface (see Figure 6). There is a local stress concentration where the load is acting and plastification starts there. The focus of this investigation was on the contour of the plasticized zone, which is far enough away from the loading and is not affected by the stress concentration there (Saint-Venant’s principle). The shape of the plastic zone is little sensitive to the element size. Even with doubled (or halved) element size there are little differences in the shape. The differences mainly concern the sharpness of the transition between purely elastic and plasticized areas, larger element size leads to smoother transitions.

## 3. Results and Discussion

### 3.1. Finite Element Analysis

**Figure 7:**
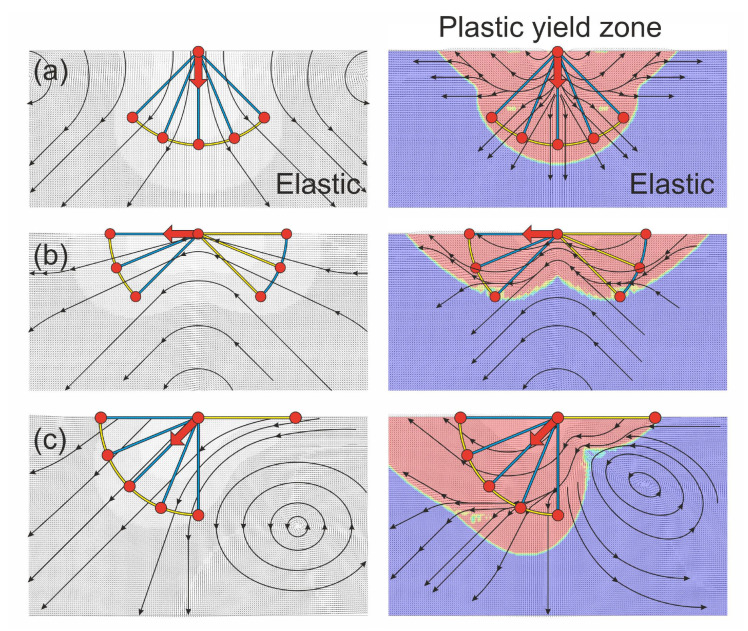
FEM results for three different forces as cross-sections at the symmetry axis. On the left-hand side, results for a purely elastic response are shown, while on the right elastic-plastic material behavior is modeled. The plastic yield zones are colored in red. In (**a**) the point force is acting perpendicular to the surface, in (**b**) the force is parallel to the surface and in (**c**) the acting force is at an angle of 45 to the surface normal. The force cone method predictions are superimposed in each panel.

The results of these FEM calculations for a purely elastic and for an elastoplastic material are presented in Figure 7 for the three loading scenarios introduced in Figure 5.

In Figure 7, the force cone method predictions are superimposed on the FEM results, which are visualized along the symmetry axes cross-sections. Comparing Figure 5 and Figure 7 demonstrates that the FEM results are in agreement with the force cone ones. There are whirl-like displacement fields in both elastic and elastoplastic calculations. The whirls are even situated as qualitatively predicted by the force cone models in Figure 5. The 2D-whirls are at both sides of the vertical force (Figure 7a), below the tangential force (Figure 7b) and on the right side under the slanted force attack (Figure 7c). Thus, there are whirls under the surface of a solid, if that surface is loaded by forces. This implies that under a tribological load, not only sliding needs to be considered, but also rolling events carried by “hidden wheels” of small amounts of rotation in the purely elastic case and larger amounts of rotation for a plastic response of the material. These “wheels” accommodate the strain induced by the shear loading. This approach to understanding the material response to a tribological load is in complete agreement with experimental results found in literature. For multilayered materials for example, vortices have been described in the literature several times [8,12] and one might even explain mechanical mixing of for instance Au-Ni multilayers [26] through the force cone approach. Such vortices have also been described for model materials such as copper [11] and simple iron carbon steels [27].

### 3.2. Simplified Model Experiments

**Figure 8:**
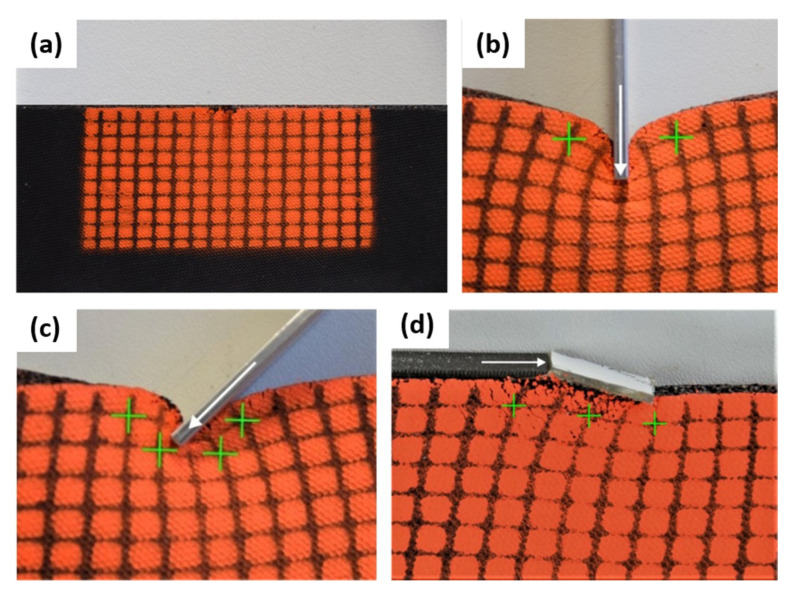
Model experiments with moss rubber. These simplified experiments illustrate the relationship between the angle of force attack and the location of the whirls. The surface of a standing moss rubber plate was coated red and marked with a rectangular grid of black lines. In (**a**) the unloaded moss rubber plate is shown. In (**b**) the surface of the rubber plate is indented vertically, in (**c**) the force is acting under an angle of 45° and (**d**) the material is loaded parallel to the surface. In (**b**–**d**), the deformed material can be followed by the grid in black, while the original—unloaded—location of select intersection point are marked by green crosses.

As both the force cone and the FEM results are in good agreement, they were put to the test experimentally. The experiments chosen are simple and had the only purpose to show that displacement whirls exist and that they are at least roughly at the predicted places. Moss rubber was used as it is softer than regular rubbers and is prone to significant deformations at relative small loads. For the experiments, depicted in Figure 8 as digital micrograph cross-sections, a little steel sheet was pushed vertically (Figure 8b) or slanted (Figure 8c) into the surface or a little plate was glued on top of the rubber and pushed tangential to the surface (Figure 8d), in order to mimic a tribological shear load.

In these experiments, the same angles for the force to act on the material were chosen as for the force cone (Figure 5) and FEM (Figure 7) considerations. At the same time one should keep in mind that here the force does not act on a single point, especially in the case of Figure 8d, where the material is loaded parallel to the surface. The rubber plate was clamped all along its lower edge. Upon following the changes on the black grid and comparing to the original locations of the intersection points between horizontal and vertical lines, it can be concluded that the maximum rotations are observed at the locations where they were predicted by the force cone method, see Figure 5. At the same time one needs to keep in mind that the large geometrically non-linear deformations of the surface do not fully agree with the theoretical calculations (both force cone method and FEM), as these assume a fully linear elastic solid. Interestingly, the rotation of the crosses formed at the intersection points between vertical and horizontal lines also are as predicted. This is further evidence that whirls exist and that they roughly behave as suggested by the force cone method. As trivial as these experiments may seem, they help to visualize that whirls are existing near where the surfaces experience a load; more should not be expected from these first experiments. In the future, it would be interesting to aim at correlating these whirls with alterations in the local mechanical properties and to investigate how these changes manifest themselves in the tribological behavior of the overall material.

In conclusion, tribologically loaded materials are known to exhibit subsurface vortices. While such structures have traditionally been associated with phenomena as they are found in fluids, we here offer an alternative explanation. The so-called force cone method, which was mainly established to create lightweight mechanical structures, allows an intuitive manner to identify the subsurface areas where compressive and tension stresses are acting. Through considering the interaction between tension and compression cones, whirl formation for forces acting perpendicular, parallel and at an angle of 45° to the surface are predicted. These results are compared to finite element calculation which themselves support the whirls predicted by the force cone method. Simple model experiments performed with moss rubber further substantiate the force cone predictions. These results suggest that “rolling instead of sliding” is a preferred mechanism to react to a shear load and that solids create “wheels” through the formation of subsurface vortices. The force cone method is an intuitive and easy to use approach to understand how solids react to shear forces, independent of the length scales that are considered. This being said, one needs to be aware that the force cone method aims at a qualitative description and so far, is not meant to arrive at quantitative predictions. Similarly, no statement is intended about the nature of the elementary mechanisms by which these whirls form or how the material accommodates plasticity.

## Figures and Tables

**Table 1:** FEM simulation parameters and geometry.

Parameter	Symbol/Description	Value
Material		
Young’s modulus (MPa)	*E*	210,000
Poisson’s ratio	*ν*	0.35
Yield strength (MPa)	*Re*	20
Plastic tangent modulus (MPa)	*E_T_*	100
Model geometry (2D)		
Width	*W*	400
Height	*H*	200
Boundary conditions		
Number of force loaded nodes	-	3
Left, right, bottom	-	Clamped
Element		
Type	Quad	PLANE 182 ^1^
Size	-	2
Number	-	20,000
Stress state	-	Plain strain

^1^ Finite element program ANSYS MECHANICAL (Canonsburg, PA, USA), Version 17.

## Data Availability

Data from this study is available from the corresponding author upon request.
